# Longitudinal dynamics of angiotensin II type 1 receptor antibodies around kidney transplantation and their impact on clinical outcomes

**DOI:** 10.3389/fimmu.2026.1960231

**Published:** 2026-10-26

**Authors:** Jayeon Ahn, Sangwan Kim, Ahram Han, Jongwon Ha, Ara Cho, Donghoon Jeong, Hajeong Lee, Yong Chul Kim, Sangil Min

**Affiliations:** 1 Department of Surgery, Seoul National University College of Medicine, Seoul, Republic of Korea; 2 Biomedical Research Institute, Seoul National University Hospital, Seoul, Republic of Korea; 3 The Transplantation Research Institute, Medical Research Center, Seoul National University College of Medicine, Seoul, Republic of Korea; 4 Department of Internal Medicine, Seoul National University Hospital, Seoul, Republic of Korea

**Keywords:** angiotensin 1 receptor antibody, antibody-mediated rejection, graft survival, kidney transplantation, screening strategy

## Abstract

**Background:**

Angiotensin II type 1 receptor antibody (AT1R-Ab) has been implicated in graft injury after kidney transplantation, but most prior studies relied on single-timepoint measurements and did not capture the antibody’s true longitudinal dynamics. This study evaluated the longitudinal trajectory of AT1R-Ab before and after kidney transplantation and its association with clinical outcomes.

**Methods:**

We conducted a single-center retrospective cohort study of 241 kidney transplant recipients with serial AT1R-Ab measurements pre-transplant, at 2 weeks, 3 months, and 1 year, along with protocol biopsies at 2 weeks and 1 year. Patients were categorized into LOW (<10 U/mL), GREY (10–17 U/mL), and HIGH (>17 U/mL) groups. Secondary outcomes included renal function, *de novo* donor-specific antibody (DSA) development, and Banff histopathology. Cox marginal structural models with inverse probability weighting were applied.

**Results:**

Among the 73 patients classified as GREY or HIGH at baseline, only 14.3% and 17.6%, respectively, remained in their baseline category throughout follow-up, with movement toward the LOW category more common than escalation to a higher category. Consistent with this, the GREY and HIGH groups did not show a significant excess risk of biopsy-proven acute rejection (BPAR) relative to the LOW group at either 1-year (aHR 0.67–1.44) or 3-year (aHR 0.69–1.17) follow-up, nor for *de novo* DSA at 1-year (aHR 1.59–2.49) or 3-year (aHR 0.89–1.14) follow-up. The initial crude signal for microvascular inflammation (MVI) at 3 years (cHR 4.92, 95% CI 1.57–15.39, p=0.006) did not persist after adjustment (aHR 2.93, 95% CI 0.97–8.85, p=0.057), a pattern also seen at 1 year (crude HR 3.08 vs. adjusted HR 2.86, p=0.063).

**Conclusion:**

Elevated AT1R-Ab levels around kidney transplantation frequently declined spontaneously, accompanied by favorable clinical outcomes—paralleling findings reported for HLA-DSA and suggesting that antibody-mediated risk may depend less on presence than on change over time. These results provide limited support for universal single-measurement screening and instead support the targeted testing strategy recommended by the 2022 STAR Working Group. Larger prospective studies are needed to confirm these findings.

## Introduction

Angiotensin II type 1 receptor antibody (AT1R-Ab) binds to the AT1 receptor, inducing microvascular inflammation and vasoconstriction, which may lead to vascular lesions such as thrombosis, endarteritis, and fibrinoid necrosis (1–5). In kidney transplantation, AT1R-Ab was first identified as a potential cause of rejection in 2005 (6) and subsequent studies reported that pre-transplant AT1R-Ab levels were significantly associated with acute rejection and long-term graft dysfunction (7).

Although AT1R-Ab has emerged as an important non-HLA antibody, its clinical use remains challenging: testing is ELISA-based and requires substantial time and cost, limiting use when rapid decisions are needed. The 2022 STAR Working Group report noted that non-HLA antibodies may play a role in transplant immunology, but that evidence for non-HLA antibody testing in suspected ABMR without HLA-DSA remains moderate (8)—implying selective rather than universal testing. Indeed, some studies report significant associations between AT1R-Ab and acute rejection (9–11) or graft survival (9, 12–14), while others find no independent association (15–19).

Much of this discrepancy likely stems from methodological limitations: arbitrarily or retrospectively defined cut-offs (7, 9); heterogeneous single pre- (9, 13–15, 19, 20) or post-transplant (10, 21) sampling, or treating any positive value as relevant regardless of timing (18); limited serial sampling leaving AT1R-Ab dynamics largely unexamined (10, 13, 14, 17, 18, 20, 21); and selection bias from cohorts restricted to rejection or graft failure (6, 21). We therefore performed repeated AT1R-Ab measurements at four predefined time points (pre-transplant, 2 weeks, 3 months, and 1 year), combined with protocol biopsy findings, to determine how AT1R-Ab levels change over time and how these patterns—particularly the spontaneous decline of initially elevated levels—relate to clinical outcomes.

## Materials and methods

### Patient population

This single-center, retrospective cohort study was conducted at tertiary academic transplant center in South Korea and included kidney transplant recipients between 2016 and 2022. Eligible patients were those who had serum samples collected at pre-transplant and at 2 weeks, 3 months, and 1-year post-transplant, with a minimum follow-up of 1 year. Exclusion criteria were simultaneous multi-organ transplantation and age younger than 18 years. Among the 1,264 patients who underwent kidney transplantation during this period, 1,117 met the eligibility criteria, of whom 349 had at least four serum samples stored in the Seoul National University Hospital Biobank within 1 year after transplantation. Of these, 105 were excluded due to the absence of required time-point samples (pre-transplant and 2-week, 3-month, and 1-year post-transplant), and an additional 3 were excluded due to missing data for studied parameters. Consequently, a total of 241 patients were included in the final analysis.

### Studied parameters

Baseline and perioperative variables collected in this study included age, sex, body mass index (BMI), the use of angiotensin receptor blockers (ARBs), hypertension, pre-transplant kidney replacement therapy (KRT), transplantation type (living vs deceased donor), donor–recipient relationship, HLA incompatibility, ABO incompatibility, desensitization therapy, biopsy results, the occurrence of *de novo* donor-specific antibodies (DSA), and post-transplant renal function parameters such as serum creatinine (SCr) and estimated glomerular filtration rate (eGFR) at 2 weeks, 3 months, and 1 year. Donor–recipient relationship was classified as related when the donor was a first- or second-degree relative, including parents, siblings, or children. Immunologic incompatibility was documented by replacing DSA positivity with HLA incompatibility. Desensitization therapy was defined as the administration of rituximab alone or in combination with plasma exchange. Desensitization was indicated for patients with pre-transplant anti-HLA donor-specific antibody (DSA) positivity or ABO incompatibility. Rituximab was administered at a fixed dose of 200 mg, and plasma exchange was continued until DSA became undetectable or T-cell flow cytometry crossmatch became negative in DSA-positive patients, or until the isoagglutinin titer decreased below 1:32 in ABO-incompatible patients. Induction immunosuppression consisted of basiliximab for ABO-incompatible patients (administered on the day of transplantation and on postoperative day 4) and anti-thymocyte globulin (total dose 4.5–7.5 mg/kg) for DSA-positive patients. All patients received standard triple maintenance immunosuppression with tacrolimus, mycophenolate mofetil, and corticosteroids. Post-transplant hypertension was classified as present if they were newly diagnosed within 1 year after transplantation or if preexisting conditions persisted during the first postoperative year. Post-transplant ARB use was categorized as “yes” when ARBs were used at any time within the first year following transplantation.

### AT1R-Ab testing

Serum samples were collected at four time points (pre-transplant, 2 weeks, 3 months, and 1-year post-transplant) and immediately stored at –80 °C. All specimens were obtained from the Seoul National University Hospital Biobank under approval from the Korean Biobank Network. AT1R-Ab levels were measured using a commercially available quantitative enzyme immunoassay (EIA) kit (One Lambda).

The analytical detection range provided by the manufacturer was 2.5–40 U/mL. Values exceeding the upper limit (>40 U/mL) were diluted for measurement; however, given the limited precision beyond this range, all values >40 U/mL were uniformly recoded as 40 U/mL to minimize measurement variability.

According to the manufacturer’s validated interpretive criteria, patients were classified at each time point into three groups: LOW (<10 U/mL), GREY (10–17 U/mL), and HIGH (>17 U/mL). These predefined cutoffs originate from the assay’s clinical reference thresholds established by the manufacturer.

### Biopsy (tissue examination)

Protocol biopsies were performed at 2 weeks and 1-year post-transplant. Indication biopsies were additionally performed when clinically warranted, such as an unexplained increase in serum creatinine (>15%), new-onset or worsening proteinuria, or based on the clinical judgment of the treating physician. All biopsy specimens were evaluated for rejection and classified according to the 2019 Banff criteria (22).

Chronicity score (23) was calculated as the sum of the following Banff chronic components: interstitial fibrosis (ci), tubular atrophy (ct), chronic glomerulopathy (cg), and vascular fibrous intimal thickening (cv). Microvascular inflammation (MVI) was defined as the sum of peritubular capillaritis (ptc) and glomerulitis (g) scores.

### Study analysis & outcome

The primary objective of this study was to characterize the longitudinal trajectory of AT1R-Ab concentrations from pre-transplant through 1 year after kidney transplantation. We then evaluated whether these temporal patterns were associated with biopsy-proven acute rejection (BPAR), *de novo* DSA development, and renal function.

The primary outcome was biopsy-proven acute rejection (BPAR), analyzed according to AT1R-Ab concentration groups. BPAR was assessed through protocol biopsies at 2 weeks and 1-year post-transplant, as well as indication biopsies when clinically required. BPAR included multiple histopathologic subtypes— T-cell–mediated rejection (TCMR), antibody-mediated rejection (ABMR), chronic active T-cell–mediated rejection (CATMR), chronic active antibody-mediated rejection (CAAMR), and hyperacute rejection. Borderline rejection was examined separately, both as part of the overall BPAR group and as an independent category.

Secondary outcomes included the incidence of *de novo* donor-specific antibody (DSA) formation and changes in renal function by AT1R-Ab group. Renal function was evaluated using serum creatinine (SCr) and estimated glomerular filtration rate (eGFR) at 2 weeks, 3 months, and 1-year post-transplant. The development of *de novo* DSA was assessed by HLA typing performed during biopsy procedures.

In addition, Banff pathological variables were analyzed in relation to AT1R-Ab levels at 1- and 3-years post-transplant. These variables included inflammation in areas of interstitial fibrosis and tubular atrophy (i-IFTA), peritubular capillaritis (PTC), chronicity score, and microvascular inflammation (MVI). This comprehensive pathological evaluation enabled correlation of antibody concentrations with histologic markers of graft injury and chronic damage.

### Statistical analysis

Group comparisons were performed at four time points: pre-transplant, and at 2 weeks, 3 months, and 1-year post-transplant. Continuous variables were summarized as means with standard deviations and compared using one-way analysis of variance (ANOVA) or Kruskal–Wallis tests, as appropriate. Categorical variables were expressed as frequencies and percentages, and differences between groups were assessed using chi-square tests or Fisher’s exact tests.

To visualize longitudinal changes in AT1R-Ab group classification, Sankey diagrams were constructed to illustrate dynamic transitions between antibody groups over time.

The associations between AT1R-Ab groups and post-transplant outcomes, including biopsy-proven acute rejection (BPAR) and *de novo* donor-specific antibody (DSA) development, were analyzed over follow-up periods of 1 and 3 years using Marginal Structural Cox Models (Cox-MSM). AT1R-Ab was modelled as a time-varying exposure: each patient contributed consecutive person-time intervals delimited by the four sampling times, and each interval was assigned the antibody group measured at its start (days 0, 14, 90 and 365). Events occurring between two measurements were attributed to the group measured at the beginning of that interval, and person-time after an event was excluded. Observations were administratively censored at the end of each follow-up window, and because serial sampling was prespecified to end at 1 year, the exposure was held constant thereafter in the 3-year analysis. Cox-MSM was used because it accommodates a time-varying exposure while balancing measured confounders across groups through inverse probability weighting.

Stabilized inverse probability weights were estimated based on the probability of remaining in a given antibody group at each time point. Covariates included the AT1R-Ab group at the preceding time point, sampling time, age, sex, body mass index (BMI), pre- and postoperative angiotensin receptor blocker (ARB) use, pre- and postoperative hypertension, preoperative diabetes, donor type, preemptive transplantation, kidney replacement therapy (KRT), donor–recipient relationship, compatibility, and desensitization.

Although the HIGH group became very small by the 1-year time point, the IPW estimates did not diverge. To prevent potential instability due to small cell sizes, stabilized weights were calculated as the product of inverse probability of treatment weighting (IPTW) and inverse probability of censoring weighting (IPCW) (24).

For secondary outcomes, boxplots were generated to visualize the distribution of eGFR and SCr at 2 weeks, 3 months, and 1-year post-transplant by AT1R-Ab group. These graphical analyses illustrated variability, medians, and outliers within each group. Statistical comparisons were performed using parametric or nonparametric tests depending on data distribution. These analyses aimed to evaluate whether higher AT1R-Ab groups were associated with impaired kidney function as reflected by eGFR or SCr levels.

Given the exploratory nature of the Banff parameter analyses and the pre-specified primary/secondary outcomes, correction for multiple comparisons was not applied.

All statistical analyses were performed using R software (version 4.5.0; R Foundation for Statistical Computing, Vienna, Austria). A two-sided p-value <0.05 was considered statistically significant.

### Ethical approval

All patients included in this study provided informed consent for the collection of human-derived materials and participation in the study. The study was approved by the Institutional Review Board (IRB No. 2410-184-1583). Clinical data for the study were collected and analyzed based on anonymized data provided by the institutional biobank.

## Results

### Demographic characteristics

Baseline characteristics across AT1R-Ab groups are summarized in **Table 1**. At the pre-transplant stage, the HIGH group had the youngest age among the three groups. The HIGH group also showed a higher proportion of living donor transplants, while the GREY and HIGH groups demonstrated higher frequencies of ABO incompatibility and HLA incompatibility compared with the LOW group. Accordingly, desensitization therapy was more frequently performed in the GREY and HIGH groups (**Table 1**).

**Table 1:** Comparison of clinical characteristics based on preoperative AT1R-Ab levels.

Characteristic	LOW (N = 168)	GREY (N = 56)	HIGH (N = 17)	p-value
AT1R-ab concentration	6.7 ± 1.5	12.8 ± 1.7	27.1 ± 8.8	< 0.001
Age	50.8 ± 12.8	45.8 ± 14.2	40.5 ± 12.9	0.001
Sex				0.260
female	57 (33.9%)	18 (32.1%)	9 (52.9%)	
male	111 (66.1%)	38 (67.9%)	8 (47.1%)	
BMI	23.3 ± 2.9	24.1 ± 3.8	22.7 ± 3.0	0.160
Preop ARB	98 (58.3%)	34 (60.7%)	8 (47.1%)	0.603
Postop ARB	66 (39.3%)	26 (46.4%)	8 (47.1%)	0.572
Preop HTN	144 (85.7%)	51 (91.1%)	15 (88.2%)	0.578
Postop HTN	106 (63.1%)	41 (73.2%)	10 (58.8%)	0.330
Donation type				0.029
living donor	135 (80.4%)	51 (91.1%)	17 (100.0%)	
deceased donor	33 (19.6%)	5 (8.9%)	0 (0.0%)	
Preop dialysis status				0.074
Preemptive	42 (25.0%)	11 (19.6%)	8 (47.1%)	
dialysis	126 (75.0%)	45 (80.4%)	9 (52.9%)	
Dialysis Type				0.160
hemodialysis(HD)	110 (65.5%)	37 (66.1%)	9 (52.9%)	
peritoneal dialysis(PD)	15 (8.9%)	6 (10.7%)	0 (0.0%)	
HD+PD	1 (0.6%)	2 (3.6%)	0 (0.0%)	
Relationship				0.178
related	79 (47.0%)	27 (48.2%)	12 (70.6%)	
unrelated	89 (53.0%)	29 (51.8%)	5 (29.4%)	
Compatibility				<0.001
compatible	142 (84.5%)	18 (32.1%)	8 (47.1%)	
ABO incompatible	24 (14.3%)	27 (48.2%)	5 (29.4%)	
HLA incompatible	2 (1.2%)	5 (8.9%)	2 (11.8%)	
ABO & HLA incompatible	0 (0.0%)	6 (10.7%)	2 (11.8%)	
Desensitization				<0.001
no	142 (84.5%)	18 (32.1%)	8 (47.1%)	
yes	26 (15.5%)	38 (67.9%)	9 (52.9%)	

BMI, Body Mass Index; preop, pre operative; postop, post operative; ARB, Angiotensin Receptor Blocker; HTN, Hypertension.

Differences in compatibility were most apparent before transplantation and at 2 weeks post-transplant but gradually diminished at 3 months and 1 year. Detailed comparisons at 2 weeks, 3 months, and 1 year are provided in the Supplementary Tables (**Supplementary Tables S1–S3**).

### Longitudinal AT1R-Ab trends

At baseline, 168 patients (69.7%) were classified into the LOW group, 56 (23.2%) into the GREY group, and 17 (7.1%) into the HIGH group, with a median AT1R-Ab concentration of 7.62 U/mL (IQR 6.01–11.26). The LOW group showed a stable longitudinal pattern, with 94.6% (159/168) maintaining LOW status throughout the entire follow-up period. In contrast, only 14.3% and 17.6% of patients in the GREY and HIGH groups, respectively, remained in the same category over time, and these two groups displayed more noticeable fluctuations in AT1R-Ab levels compared with the LOW group, with most transitions showing a tendency toward movement into the LOW category rather than escalation to higher levels (**Figure 1**). Overall, antibody level changes over time were more frequently observed among patients initially classified as GREY or HIGH, whereas those in the baseline LOW group largely maintained their status without major fluctuations.

**Figure 1:**
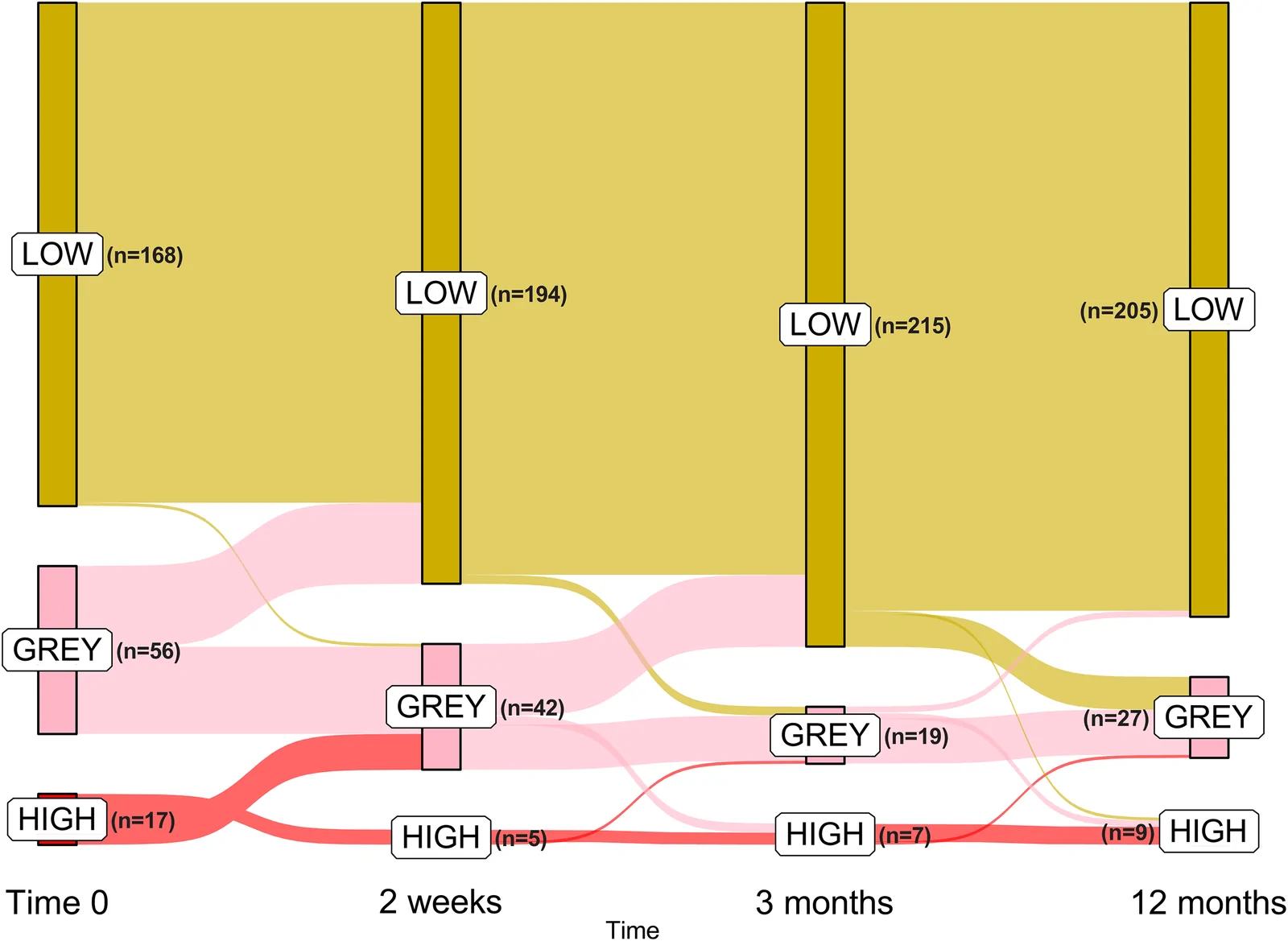
Changes in AT1R-Ab levels over time: Sankey diagram. Sankey diagram showing longitudinal transitions among LOW (<10 U/mL), GREY (10–17 U/mL), and HIGH (>17 U/mL) AT1R-Ab groups from pre-transplant to 1 year post-transplant. Band width indicates the number of patients in each transition, with a predominant shift toward the LOW category over time.

### Analysis of the association between AT1R-Ab Level and BPAR and Banff parameters

Protocol biopsies were performed in 230 patients at 2 weeks and in 209 patients at 1-year post-transplant. Two patients did not undergo biopsy, and the remainder received either protocol or indication biopsies. BPAR was evaluated up to 3 years post-transplant, and analyses were conducted both including and excluding borderline rejection. To control for confounders, we applied Cox-MSM.

When borderline rejection was included, BPAR occurred in 90 patients within 1 year and in 111 patients within 3 years, with mean observation periods of 62.7 ± 112.9 days and 150.2 ± 228.0 days, respectively. Additionally, Antibody-Mediated Rejection (ABMR) occurred in 11 patients within 1 year (1 patient experienced ABMR twice within 1 year) and in 14 patients within 3 years (4 patients developed ABMR between 1 and 3 years, and one of these had ABMR within the first year as well). In the Cox-MSM analysis, no significant association was found between AT1R-Ab groups and the risk of BPAR at either time point.

The adjusted hazard ratios (aHR) for biopsy-proven acute rejection (BPAR) at 1-year and 3-year follow-ups were as follows: for the GREY group, aHR was 0.67 (95% CI: 0.47–1.22) at 1 year and 0.69 (95% CI: 0.40–1.18) at 3 years; for the HIGH group, aHR was 1.44 (95% CI: 0.64–3.28) at 1 year and 1.17 (95% CI: 0.51–2.67) at 3 years (**Table 2**). No significant associations were observed between the AT1R-Ab groups and BPAR risk at either follow-up time point.

**Table 2:** Analysis of biopsy proven acute rejection (BPAR) (including borderline rejection) occurrence by AT1R-Ab concentration groups.

Follow-up period	Variable		HR(95% CI)	p-value
1-Year	Crude	LOW	Ref.	
		GREY	0.69 (0.40, 1.20)	0.192
		HIGH	1.16 (0.53, 2.50)	0.713
	Adjusted	LOW	Ref.	
		GREY	0.67 (0.47, 1.22)	0.188
		HIGH	1.44 (0.64, 3.28)	0.380
3-Year	Crude	LOW	Ref.	
		GREY	0.73 (0.42, 1.28)	0.277
		HIGH	1.46 (0.67, 3.18)	0.343
	Adjusted	LOW	Ref.	
		GREY	0.69 (0.40, 1.18)	0.173
		HIGH	1.17 (0.51, 2.67)	0.708

HR, Hazard Ratio; CI, Confidence Intervals.

When borderline rejection was excluded, similar results were observed. For the GREY group, the aHR at 1 year was 1.54 (95% CI: 0.69–3.44), and at 3 years, it was 1.42 (95% CI: 0.68–2.97). For the HIGH group, the aHR at 1 year was 2.13 (95% CI: 0.59–7.68), and at 3 years, it was 1.57 (95% CI: 0.44–5.53) (**Supplementary Table S4**). No significant associations were observed in either follow-up time point.

After adjustment, Banff parameter analysis revealed no significant differences among AT1R-Ab groups for i-IFTA or chronicity score at either 1-year or 3-year follow-up (**Supplementary Tables S5, S7**). However, peritubular capillaritis (PTC) was significantly higher in the HIGH group after adjustment at both 1 year (aHR 4.43, 95% CI 1.55–12.67, p=0.005) and 3 years (aHR 7.49, 95% CI 2.40–23.40, p=0.001), whereas the GREY group did not differ significantly from LOW at either time point (**Supplementary Table S6**). At 3 years post-transplant, MVI was observed in 35.3% (6/17) of patients in the HIGH group, 46.4% (26/56) in the GREY group, and 26.8% (45/168) in the LOW group. Although MVI was more frequent in the HIGH group, the crude Cox model revealed a significant association at both 1 year (cHR 3.08, 95% CI 1.05–9.03, p = 0.040) and 3 years (cHR 4.92, 95% CI 1.57–15.39, p = 0.006), neither of which remained significant after adjustment (aHR 2.86, 95% CI 0.94–8.65, p=0.063 at 1 year; aHR 2.93, 95% CI 0.97–8.85, p=0.057 at 3 years) (**Table 3**).

**Table 3:** Analysis of MVI (Microvascular inflammation) by AT1R-Ab concentration groups at 1-year and 3-year follow-up.

Follow-up period	Variable		HR(95% CI)	p-value
1-Year	Crude	LOW	Ref.	
		GREY	1.04 (0.39, 2.78)	0.939
		HIGH	3.08 (1.05, 9.03)	0.040
	Adjusted	LOW	Ref.	
		GREY	1.00 (0.38, 2.68)	0.994
		HIGH	2.86 (0.94, 8.65)	0.063
3-Year	Crude	LOW	Ref.	
		GREY	1.48 (0.52, 4.18)	0.462
		HIGH	4.92 (1.57, 15.39)	0.006
	Adjusted	LOW	Ref.	
		GREY	1.03 (0.38, 2.76)	0.958
		HIGH	2.93 (0.97, 8.85)	0.057

HR, Hazard Ratio; CI, Confidence Intervals.

### Analysis of the relationship between *de novo* DSA incidence and AT1R-Ab levels

*De novo* DSA developed in 16 patients within 1 year, with a mean observation period of 87.3 ± 109.9 days, and in 27 patients within 3 years, with a mean observation period of 358.7 ± 367.8 days. We employed a Cox MSM with IPW to evaluate the association between AT1R-Ab groups and the risk of *de novo* DSA development.

In the Cox MSM analysis, no significant associations were observed between AT1R-Ab groups and the risk of *de novo* DSA at either time point. The aHR and 95% CI were as follows: 1-year follow-up –GREY aHR 1.59 (95% CI 0.41–6.14) and HIGH aHR 2.49 (95% CI 0.28–22.01); 3-year follow-up – GREY aHR 0.89 (95% CI 0.26–3.07) and HIGH aHR 1.14 (95% CI 0.14–9.28) (**Table 4**). No statistically significant differences were found either before or after adjustment.

**Table 4:** Analysis of *de novo* DSA incidence by AT1R-Ab concentration groups at 1-year and 3-year follow-up.

Follow-up period	Variable		HR(95% CI)	p-value
1-Year	Crude	LOW	Ref.	
		GREY	1.00 (0.35, 2.90)	0.996
		HIGH	0.90 (0.12, 6.65)	0.917
	Adjusted	LOW	Ref.	
		GREY	1.59 (0.41, 6.14)	0.503
		HIGH	2.49 (0.28, 22.01)	0.413
3-Year	Crude	LOW	Ref.	
		GREY	0.97 (0.33, 2.81)	0.954
		HIGH	2.17 (0.28, 17.07)	0.461
	Adjusted	LOW	Ref.	
		GREY	0.89 (0.26, 3.07)	0.855
		HIGH	1.14 (0.14, 9.28)	0.900

HR, Hazard Ratio; CI, Confidence Intervals.

### Trends in renal function by AT1R-Ab level at different time points

When patients were categorized into LOW, GREY, and HIGH groups based on pre-transplant AT1R-Ab levels, no significant differences in renal function were observed between the three groups at any of the time points (**Figures 2A, B**). When patients were instead classified by their AT1R-Ab levels at 2 weeks and 3 months, renal function likewise showed no significant between-group differences (**Supplementary Tables S1, S2**). However, when patients were classified by their AT1R-Ab levels at 1 year (**Supplementary Table S3**), eGFR differed significantly between groups, whereas serum creatinine did not.

**Figure 2:**
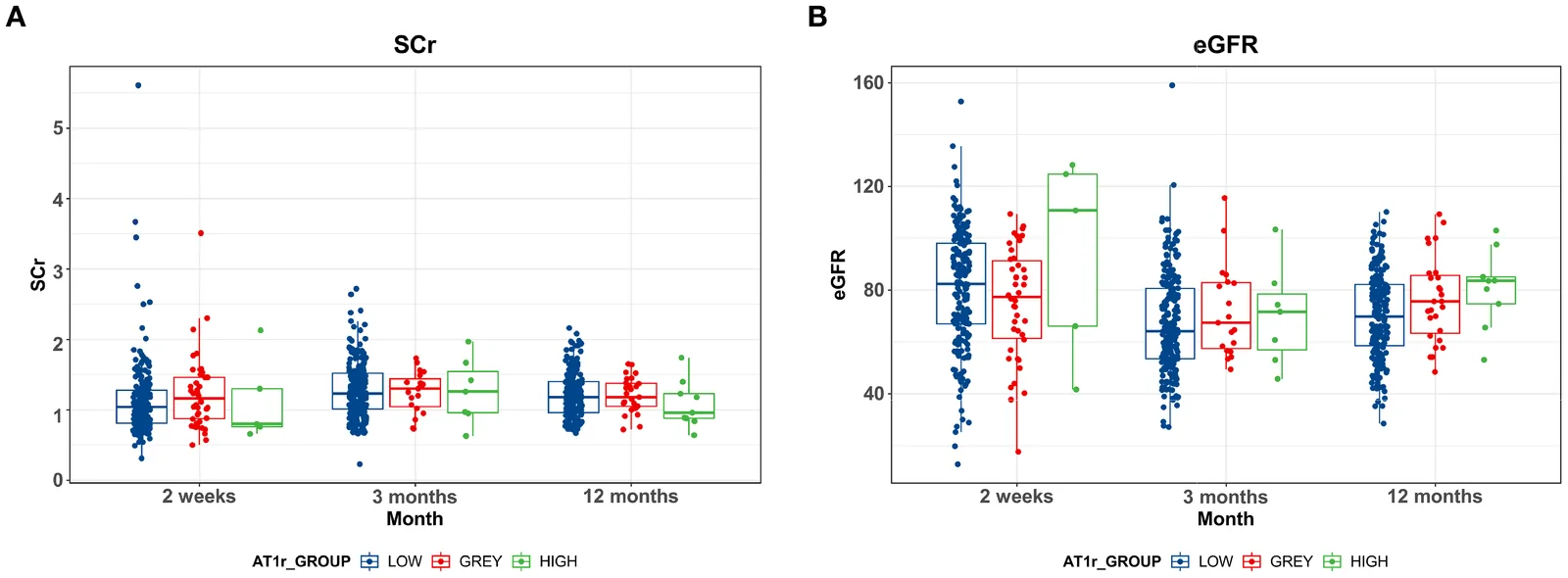
Changes in serum creatinine (SCr) and estimated glomerular filtration rate (eGFR) by AT1R-Ab concentration group at 2 weeks, 3 months, and 1 year post-transplant. **(A)** Distribution of SCr by AT1R-Ab concentration group (LOW, GREY, HIGH) at 2 weeks, 3 months, and 1 year post-transplant. Box plots comparing serum creatinine distributions across LOW, GREY, and HIGH AT1R-Ab groups at three post-transplant time points, showing overlapping distributions with no significant between-group difference. **(B)** Distribution of eGFR by AT1R-Ab concentration group (LOW, GREY, HIGH) at 2 weeks, 3 months, and 1 year post-transplant. Box plots comparing eGFR distributions across LOW, GREY, and HIGH AT1R-Ab groups at three post-transplant time points, showing overlapping distributions with no significant between-group difference.

## Discussion

This study performed repeated AT1R-Ab measurements at four time points from before to 1 year after kidney transplantation to characterize its longitudinal trajectory, and evaluated associations with clinical outcomes including BPAR, *de novo* DSA, renal function, and MVI. Among the 73 patients classified as GREY or HIGH at baseline, only 14.3% and 17.6%, respectively, remained in their baseline category throughout follow-up, with movement toward LOW more common than escalation. Consistent with this, GREY and HIGH groups showed no significant excess risk of BPAR, *de novo* DSA, or renal dysfunction relative to LOW, and the initial MVI signal did not persist after adjustment. A significant eGFR difference was observed only in the exploratory analysis classifying patients by their concurrent AT1R-Ab group at 1 year; however, this finding was not accompanied by a corresponding difference in serum creatinine and should be interpreted cautiously given the small HIGH-group sample size and multiple comparisons.

This pattern—elevated antibody levels resolving spontaneously rather than persisting, with resolution accompanied by favorable outcomes—parallels the HLA-DSA literature, where spontaneously resolved or declining-MFI *de novo* DSA predicts superior graft survival (25) and resolved preformed DSA carries a prognosis similar to DSA-negative patients (26). These prior studies were conducted exclusively in HLA-DSA; our findings extend this pattern to AT1R-Ab, suggesting that non-HLA antibody significance may likewise depend less on presence than on change over time.

Dragun et al. proposed a mechanism whereby AT1R-Ab induces vascular inflammation, based on steroid-resistant rejection cohorts (6) that limit generalizability. Subsequent studies reported inconsistent associations between AT1R-Ab and outcomes such as ABMR (12), graft failure (7), and adverse events broadly (9–14, 20–22, 27), while others found no significant correlation (15–19, 28), leaving its clinical significance uncertain. Given its association with *de novo* FSGS (29) and lower tacrolimus levels in AT1R-Ab-positive patients (30), AT1R-Ab may act as a cofactor in high-risk immunological settings rather than an isolated pathogenic factor.

As outlined in the Introduction, much of this inconsistency stems from single-time-point study designs—a limitation explicitly acknowledged even in recent work (31). Our longitudinal design directly addresses this gap. Supporting this, Min et al. found that AT1R-Ab was associated with MVI only in indication biopsies, whereas in protocol biopsies, DSA remained the sole risk factor (13). These observations suggest that the role of AT1R-Ab may differ depending on clinical context.

This study addressed several methodological challenges in prior AT1R-Ab research. We applied cut-off values of 10 U/mL (7, 10, 13, 15, 27) and 17 U/mL (11, 16–18, 20), consistent with manufacturer-recommended thresholds; however, these may not fully capture clinically optimized transplant-specific thresholds. We also restricted inclusion to patients with measurements at all four time points, enabling temporal evaluation but potentially introducing selection bias—reflecting our aim of characterizing kinetics in a clinically stable cohort rather than a limitation to be corrected, and may explain the small HIGH group. Given this limited size, the wide confidence intervals observed—particularly for the 3-year *de novo* DSA analysis (aHR 1.14, 95% CI 0.14–9.28)—indicate that the absence of statistical significance should not be interpreted as evidence of no clinically meaningful association; rather, these null findings are hypothesis-generating rather than definitive, warranting confirmation in larger cohorts. In addition, although pre-transplant HLA incompatibility—used as a proxy for donor-specific antibody (DSA) positivity—was included as a covariate, quantitative DSA titers were not available, precluding a more detailed analysis of the interaction between DSA and AT1R-Ab. Proteinuria was also not systematically measured at all study time points in this cohort and could therefore not be incorporated into the renal function analysis; future studies should consider including it alongside eGFR to more comprehensively assess AT1R-Ab’s impact on graft function.

The predominance of living-donor transplantation in our cohort (84.2%, 203/241) reflects the general landscape of kidney transplantation in South Korea, where deceased-donor transplantation remains comparatively underdeveloped and living donation constitutes the majority of transplants nationwide; nationally, 64.8% of kidney transplants are performed using living donors (32). The higher proportion in our single-center cohort may additionally reflect referral patterns at a tertiary center with an active living-donor and ABO/HLA-incompatible transplant program. As this donor composition is a structural feature of the Korean transplant system rather than a cohort-specific selection bias, it is unlikely to have materially distorted our internal findings; however, it may limit the generalizability of our results to populations with a higher proportion of deceased-donor transplantation.

From a clinical perspective, routine AT1R-Ab measurement in unselected recipients is unlikely to provide meaningful prognostic information: associations with rejection, MVI, or graft dysfunction were not reproduced once temporal dynamics and confounding were accounted for. One notable exception was PTC, which remained significantly elevated in the HIGH group after adjustment at both 1 and 3 years; however, given the very small size of the HIGH group and the lack of a concordant signal in other outcomes such as MVI, this isolated finding warrants confirmation in larger cohorts rather than immediate clinical interpretation. Overall, AT1R-Ab does not appear to independently drive allograft injury in the general population. Its value may instead lie in narrower contexts—suspected antibody-mediated rejection without HLA-DSA, recurrent glomerulonephritis, or multiple prior rejections—where it may aid immunologic assessment. Confirming this requires large, multicenter studies; our longitudinal findings may help clarify AT1R-Ab’s significance in the interim.

Elevated AT1R-Ab levels around kidney transplantation frequently declined spontaneously, and this resolution was accompanied by favorable clinical outcomes. This pattern, observed here in AT1R-Ab—a non-HLA antibody—parallels findings reported for HLA-DSA, suggesting that the clinical significance of antibody-mediated risk may depend less on the mere presence of the antibody than on how it changes over time. These results provide limited support for universal screening based on a single measurement and instead support the targeted, rather than universal, testing strategy recommended by the 2022 STAR Working Group. Larger prospective studies are needed to confirm these findings.

## Data Availability

The datasets generated and analyzed in this study contain individual patient-level clinical information obtained from the institutional biobank and medical records. Because of patient privacy and the terms of participants' consent, these data cannot be made publicly available. Requests for access for academic research purposes only may be directed to the corresponding author and will be considered subject to approval by the Institutional Review Board of Seoul National University Hospital and applicable ethical and legal requirements.

## References

[1] Sorohan BM Ismail G Leca N Tacu D Obrișcă B Constantinescu I . Angiotensin II type 1 receptor antibodies in kidney transplantation: an evidence-based comprehensive review. Transplant Rev (Orlando). (2020) 34:100573. doi: 10.1016/j.trre.2020.100573 33002671

[2] Lee H Oh E . Angiotensin II type 1 receptor antibodies in kidney transplantation. Clin Transplant Res. (2019) 33:6–12. doi: 10.4285/jkstn.2019.33.1.6

[3] Kang ZY Liu C Liu W Li DH . Effect of anti-angiotensin II type 1 receptor antibodies on the outcomes of kidney transplantation: a systematic review and meta-analysis. Nephrol Dial Transplant. (2022) 37:1171–80. doi: 10.1093/ndt/gfab344 34865146

[4] Viboon S Townamchai N Phiancharoen S Kupatawintu P Dhitivat V Nathalang O . Prevalence of antibody against angiotensin II type 1 receptor (AT1R) among Thai kidney transplant patients. Transplant Proc. (2018) 50:1310–5. doi: 10.1016/j.transproceed.2018.03.020 29880351

[5] Reinsmoen NL . Role of angiotensin II type 1 receptor-activating antibodies in solid organ transplantation. Hum Immunol. (2013) 74:1474–7. doi: 10.1016/j.humimm.2013.06.034 23831255

[6] Dragun D Müller DN Bräsen JH Fritsche L Nieminen-Kelhä M Dechend R . Angiotensin II type 1-receptor activating antibodies in renal-allograft rejection. N Engl J Med. (2005) 352:558–69. doi: 10.1056/NEJMoa035717 15703421

[7] Giral M Foucher Y Dufay A Duong Van Huyen JP Renaudin K Moreau A . Pretransplant sensitization against angiotensin II type 1 receptor is a risk factor for acute rejection and graft loss. Am J Transplant. (2013) 13:2567–76. doi: 10.1111/ajt.12397 23919486

[8] Tambur AR Bestard O Campbell P Chong AS Barrio MC Ford ML . Sensitization in transplantation: assessment of risk 2022 working group meeting report. Am J Transplant. (2023) 23:133–49. doi: 10.1016/j.ajt.2022.11.009 36695615

[9] Banasik M Boratyńska M Kościelska-Kasprzak K Kamińska D Bartoszek D Zabińska M . The influence of non-HLA antibodies directed against angiotensin II type 1 receptor (AT1R) on early renal transplant outcomes. Transpl Int. (2014) 27:1029–38. doi: 10.1111/tri.12371 24909812

[10] Lefaucheur C Viglietti D Bouatou Y Philippe A Pievani D Aubert O . Non-HLA agonistic anti-angiotensin II type 1 receptor antibodies induce a distinctive phenotype of antibody-mediated rejection in kidney transplant recipients. Kidney Int. (2019) 96:189–201. doi: 10.1016/j.kint.2019.01.030 31005275

[11] Liu C Kang ZY Yin Z Xiao Y Liu W Zhao Y . Levels of angiotensin II type-1 receptor antibodies and endothelin-1 type-A receptor antibodies correlate with antibody-mediated rejection and poor graft function in kidney-transplantation patients. Transpl Immunol. (2022) 74:101674. doi: 10.1016/j.trim.2022.101674 35901953

[12] Taniguchi M Rebellato LM Cai J Hopfield J Briley KP Haisch CE . Higher risk of kidney graft failure in the presence of anti-angiotensin II type-1 receptor antibodies. Am J Transplant. (2013) 13:2577–89. doi: 10.1111/ajt.12395 23941128

[13] Min JW Lee H Choi BS Park CW Yang CW Kim YS . Clinical impact of pre-transplant antibodies against angiotensin II type I receptor and major histocompatibility complex class I-related chain A in kidney transplant patients. Ann Lab Med. (2018) 38:450–7. doi: 10.3343/alm.2018.38.5.450 29797816 PMC5973920

[14] Cuevas E Arreola-Guerra JM Hernández-Méndez EA Salcedo I Castelán N Uribe-Uribe NO . Pretransplant angiotensin II type 1-receptor antibodies are a risk factor for earlier detection of de novo HLA donor-specific antibodies. Nephrol Dial Transplant. (2016) 31:1738–45. doi: 10.1093/ndt/gfw204 27220757

[15] Filiopoulos V Vittoraki A Vallianou K Bellos I Markaki P Liapis G . The influence of antibodies against angiotensin II type-1 receptor on the outcome of kidney transplantation: a single-center retrospective study. J Clin Med. (2023) 12:3112. doi: 10.3390/jcm12093112 37176553 PMC10179262

[16] Pinelli DF Friedewald JJ Haarberg KMK Radhakrishnan SL Zitzner JR Hanshew WE . Assessing the potential of angiotensin II type 1 receptor and donor specific anti-endothelial cell antibodies to predict long-term kidney graft outcome. Hum Immunol. (2017) 78:421–7. doi: 10.1016/j.humimm.2017.03.012 28372986

[17] Pizzo H Mirocha J Choi J Garrison J Haas M Zhang X . Pre-transplant angiotensin II receptor type I antibodies in pediatric renal transplant recipients: an observational cohort study. Pediatr Transplant. (2022) 26:e14400. doi: 10.1111/petr.14400 36168673

[18] Aljishi M Isbel NM Jegatheesan D Johnson DW Cho Y Campbell SB . Rejection and graft outcomes in kidney transplant recipients with and without angiotensin II receptor type 1 antibodies. Transpl Immunol. (2023) 76:101756. doi: 10.1016/j.trim.2022.101756 36460263

[19] Deltombe C Gillaizeau F Anglicheau D Morelon E Trébern-Launay K Le Borgne F . Is pre-transplant sensitization against angiotensin II type 1 receptor still a risk factor of graft and patient outcome in kidney transplantation in the anti-HLA Luminex era? A retrospective study. Transpl Int. (2017) 30:1150–60. doi: 10.1111/tri.13009 28686316

[20] Philogene MC Zhou S Lonze BE Bagnasco S Alasfar S Montgomery RA . Pre-transplant screening for non-HLA antibodies: who should be tested? Hum Immunol. (2018) 79:195–202. doi: 10.1016/j.humimm.2018.02.001 29428484

[21] Lee H Kim JI Moon IS Chung BH Yang CW Kim Y . Investigation of serum angiotensin II type 1 receptor antibodies at the time of renal allograft rejection. Ann Lab Med. (2015) 35:314–20. doi: 10.3343/alm.2015.35.3.314 25932439 PMC4390699

[22] Loupy A Haas M Roufosse C Naesens M Adam B Afrouzian M . The Banff 2019 Kidney Meeting Report (I): updates on and clarification of criteria for T cell- and antibody-mediated rejection. Am J Transplant. (2020) 20:2318–31. doi: 10.1111/ajt.15898 32463180 PMC7496245

[23] Panzer SE Joachim E Parajuli S Zhong W Astor BC Djamali A . Glomerular C3 deposition is an independent risk factor for allograft failure in kidney transplant recipients with transplant glomerulopathy. Kidney Int Rep. (2019) 4:582–93. doi: 10.1016/j.ekir.2019.01.018 30993233 PMC6451156

[24] Robins JM Hernán MA Brumback B . Marginal structural models and causal inference in epidemiology. Epidemiology. (2000) 11:550–60. doi: 10.1097/00001648-200009000-00011 10955408

[25] López Del Moral C Wu K Naik M Osmanodja B Akifova A Lachmann N . The natural history of de novo donor-specific HLA antibodies after kidney transplantation. Front Med (Lausanne). (2022) 9:943502. doi: 10.3389/fmed.2022.943502 36186822 PMC9523126

[26] Gniewkiewicz M Czerwinska K Zielniok K Durlik M . Impact of resolved preformed, persistent preformed, and de novo anti-HLA donor-specific antibodies in kidney transplant recipients on long-term renal graft outcomes. J Clin Med. (2023) 12:3361. doi: 10.3390/jcm12103361 37240467 PMC10219016

[27] Sorohan BM Ismail G Berechet A Obrișcă B Constantinescu I Mărunțelu I . The early impact of preformed angiotensin II type 1 receptor antibodies on graft function in a low immunological risk cohort of kidney transplant recipients. Transpl Immunol. (2021) 66:101389. doi: 10.1016/j.trim.2021.101389 33838295

[28] Harmacek D Weidmann L Rho E Castrezana Lopez K Westphal F Nilsson J . Non-human leukocyte antigen autoantibodies-anti-angiotensin II type 1 receptor and endothelin type A receptor antibodies-in the diagnosis of antibody-mediated rejection: biopsy-based transcript diagnostics perspective. Am J Transplant. (2025) 25:2320–8. doi: 10.1016/j.ajt.2025.07.2476 40738402

[29] Abuzeineh M Aala A Alasfar S Alachkar N . Angiotensin II receptor 1 antibodies associate with post-transplant focal segmental glomerulosclerosis and proteinuria. BMC Nephrol. (2020) 21:253. doi: 10.1186/s12882-020-01910-w 32615995 PMC7331243

[30] Sorohan BM Sinescu I Tacu D Bucsa C Tincu C Obrișcă B . Immunosuppression as a risk factor for de novo angiotensin II type receptor antibodies development after kidney transplantation. J Clin Med. (2021) 10:5390. doi: 10.3390/jcm10225390 34830672 PMC8625545

[31] Mizera J Marek-Bukowiec K Moll G Catar R Heidecke H Schulze-Forster K . Anti-angiotensin II type 1 receptor autoantibodies of IgG3 subclass outperform total anti-AT1R IgG1-IgG4 levels in predicting transplanted kidney antibody-mediated rejection. Front Immunol. (2026) 17:1776113. doi: 10.3389/fimmu.2026.1776113 41846918 PMC12989333

[32] Jeon HJ Koo TY Ju MK Chae DW Choi SJN Kim MS . The Korean Organ Transplantation Registry (KOTRY): an overview and summary of the kidney-transplant cohort. Kidney Res Clin Pract. (2022) 41:492–507. doi: 10.23876/j.krcp.21.185 35919926 PMC9346403

