# Supplementary material for: Longitudinal dynamics of angiotensin II type 1 receptor antibodies around kidney transplantation and their impact on clinical outcomes
Source: Front Immunol. 2026 Oct 26;17:1960231. doi: 10.3389/fimmu.2026.1960231 (PMC13745487; doi:10.3389/fimmu.2026.1960231)
Supplement: Supplementary file 1 [file DataSheet1.pdf]

Supplement Table 1. Comparison of Clinical Characteristics Based on AT1R-Ab Levels at 2 Weeks Postoperative

	LOW(N=194)	GREY(N=42)	HIGH(N=5)	p-value
AT1R-ab concentration	6.0 ± 1.7	12.2 ± 1.7	30.97 ± 6.0	<0.001
SCr at 2 weeks	1.1 ± 0.6	1.2 ± 0.5	1.1 ± 0.6	0.535
eGFR at 2 weeks	80.3 ± 23.0	74.9 ± 21.5	94.3 ± 38.5	0.141
Age	50.4 ± 13.1	44.1 ± 13.2	31.2 ± 12.1	<0.001
Sex				0.856
female	66 (34.0%)	16 (38.1%)	2 (40.0%)	
male	128 (66.0%)	26 (61.9%)	3 (60.0%)	
BMI	23.4 ± 3.0	24.1 ± 3.8	22.3 ± 2.8	0.299
Preop ARB	113 (58.2%)	25 (59.5%)	2 (40.0%)	0.701
Postop ARB	80 (41.2%)	18 (42.9%)	2 (40.0%)	0.979
Preop HTN	168 (86.6%)	38 (90.5%)	4 (80.0%)	0.706
Postop HTN	125 (64.4%)	28 (66.7%)	4 (80.0%)	0.751
Donation type				0.017
living donor	157 (80.9%)	41 (97.6%)	5 (100.0%)	
deceased donor	37 (19.1%)	1 (2.4%)	0 (0.0%)	
Preop dialysis status				0.195
Preemptive	48 (24.7%)	10 (23.8%)	3 (60.0%)	
dialysis	146 (75.3%)	32 (76.2%)	2 (40.0%)	
Dialysis Type				0.201
hemodialysis(HD)	128 (66.0%)	26 (61.9%)	2 (40.0%)	
peritoneal dialysis(PD)	17 (8.8%)	4 (9.5%)	0 (0.0%)	
HD+PD	1 (0.5%)	2 (4.8%)	0 (0.0%)	
Relationship				0.029
related	89 (45.9%)	24 (57.1%)	5 (100.0%)	
unrelated	105 (54.1%)	18 (42.9%)	0 (0.0%)	
Compatibility				<0.001
compatible	149 (76.8%)	15 (35.7%)	4 (80.0%)	
ABO incompatible	36 (18.6%)	19 (45.2%)	1 (20.0%)	
HLA incompatible	6 (3.1%)	3 (7.1%)	0 (0.0%)	
ABO & HLA incompatible	3 (1.5%)	5 (11.9%)	0 (0.0%)	
Desensitization				<0.001
no	149 (76.8%)	15 (35.7%)	4 (80.0%)	
yes	45 (23.2%)	27 (64.3%)	1 (20.0%)	

SCr; Serum Creatinine, eGFR; Estimated Glomerular Filtration Rate, BMI; Body Mass Index, preop; pre operative, postop; post operative, ARB; Angiotensin Receptor Blocker, HTN; Hypertension

Supplement Table 2. Comparison of Clinical Characteristics Based on AT1R-Ab Levels at 3 Months Postoperative

	LOW(N=215)	GREY(N=19)	HIGH(N=7)	p-value
AT1R-ab concentration	5.9 ± 1.6	12.2 ± 1.6	23.91 ± 7.7	<0.001
SCr at 3 months	1.3 ± 0.4	1.2 ± 0.3	1.3 ± 0.5	0.916
eGFR at 3 months	67.2 ± 19.4	71.9 ± 17.9	70.2 ± 19.4	0.563
Age	50.2 ± 13.0	38.9 ± 13.0	35.3 ± 13.0	<0.001
Sex				0.44
female	73 (34.0%)	7 (36.8%)	4 (57.1%)	
male	142 (66.0%)	12 (63.2%)	3 (42.9%)	
BMI	23.5 ± 3.0	23.7 ± 4.2	21.1 ± 3.5	0.125
Preop ARB	125 (58.1%)	14 (73.7%)	1 (14.3%)	0.025
Postop ARB	90 (41.9%)	6 (31.6%)	4 (57.1%)	0.475
Preop HTN	187 (87.0%)	17 (89.5%)	6 (85.7%)	0.946
Postop HTN	140 (65.1%)	12 (63.2%)	5 (71.4%)	0.926
Donation_type				0.199
living donor	178 (82.8%)	18 (94.7%)	7 (100.0%)	
deceased donor	37 (17.2%)	1 (5.3%)	0 (0.0%)	
Preop dialysis status				0.251
Preemptive	51 (23.7%)	7 (36.8%)	3 (42.9%)	
dialysis	164 (76.3%)	12 (63.2%)	4 (57.1%)	
Dialysis Type				0.005
hemodialysis(HD)	144 (67.0%)	8 (42.1%)	4 (57.1%)	
peritoneal dialysis(PD)	19 (8.8%)	2 (10.5%)	0 (0.0%)	
HD+PD	1 (0.5%)	2 (10.5%)	0 (0.0%)	
Relationship				0.001
related	96 (44.7%)	16 (84.2%)	6 (85.7%)	
unrelated	119 (55.3%)	3 (15.8%)	1 (14.3%)	
Compatible				0.863
Compatibility	150 (69.8%)	12 (63.2%)	6 (85.7%)	
ABO incompatible	49 (22.8%)	6 (31.6%)	1 (14.3%)	
HLA incompatible	8 (3.7%)	1 (5.3%)	0 (0.0%)	
ABO & HLA incompatible	8 (3.7%)	0 (0.0%)	0 (0.0%)	
Desensitization				0.539
no	150 (69.8%)	12 (63.2%)	6 (85.7%)	
yes	65 (30.2%)	7 (36.8%)	1 (14.3%)	

SCr; Serum Creatinine, eGFR; Estimated Glomerular Filtration Rate, BMI; Body Mass Index, preop; pre operative, postop; post operative, ARB; Angiotensin Receptor Blocker, HTN; Hypertension

Supplement Table 3. Comparison of Clinical Characteristics Based on AT1R-Ab Levels at 1 Year Postoperative

	LOW(N=205)	GREY(N=27)	HIGH(N=9)	p-value
AT1R-ab concentration	5.8 ± 1.5	12.2 ± 1.8	26.93 ± 9.6	<0.001
SCr at 1 year	1.2 ± 0.3	1.2 ± 0.2	1.1 ± 0.3	0.488
eGFR at 1 year	70.1 ± 16.8	76.4 ± 16.6	80.8 ± 15.2	0.043
Age	51.2 ± 12.3	35.3 ± 13.1	38.1 ± 12.3	<0.001
Sex				0.412
female	70 (34.1%)	9 (33.3%)	5 (55.6%)	
male	135 (65.9%)	18 (66.7%)	4 (44.4%)	
BMI	23.5 ± 3.0	24.0 ± 4.0	22.2 ± 3.6	0.328
Preop ARB	122 (59.5%)	16 (59.3%)	2 (22.2%)	0.085
Postop ARB	86 (42.0%)	10 (37.0%)	4 (44.4%)	0.873
Preop HTN	179 (87.3%)	23 (85.2%)	8 (88.9%)	0.941
Postop HTN	135 (65.9%)	16 (59.3%)	6 (66.7%)	0.792
Donation_type				0.405
living donor	171 (83.4%)	23 (85.2%)	9 (100.0%)	
deceased donor	34 (16.6%)	4 (14.8%)	0 (0.0%)	
Preop dialysis status				0.115
Preemptive	47 (22.9%)	11 (40.7%)	3 (33.3%)	
dialysis	158 (77.1%)	16 (59.3%)	6 (66.7%)	
Dialysis Type				0.384
hemodialysis(HD)	138 (67.3%)	13 (48.1%)	5 (55.6%)	
peritoneal dialysis(PD)	18 (8.8%)	2 (7.4%)	1 (11.1%)	
HD+PD	2 (1.0%)	1 (3.7%)	0 (0.0%)	
Relationship				<0.001
related	89 (43.4%)	22 (81.5%)	7 (77.8%)	
unrelated	116 (56.6%)	5 (18.5%)	2 (22.2%)	
Compatibility				0.783
compatible	143 (69.8%)	19 (70.4%)	6 (66.7%)	
ABO incompatible	47 (22.9%)	6 (22.2%)	3 (33.3%)	
HLA incompatible	7 (3.4%)	2 (7.4%)	0 (0.0%)	
ABO & HLA incompatible	8 (3.9%)	0 (0.0%)	0 (0.0%)	
Desensitization				0.978
no	143 (69.8%)	19 (70.4%)	6 (66.7%)	
yes	62 (30.2%)	8 (29.6%)	3 (33.3%)	

SCr; Serum Creatinine, eGFR; Estimated Glomerular Filtration Rate, BMI; Body Mass Index, preop; pre operative, postop; post operative, ARB; Angiotensin Receptor Blocker, HTN; Hypertension

Supplement Table 4. Analysis of Biopsy Proven Acute Rejection (BPAR) (excluding Borderline Rejection) Occurrence by AT1R-Ab Concentration Groups

Follow-up period	Variable		HR(95% CI)	p-value
1-Year	Crude	LOW	Ref.	
		GREY	1.42 (0.66, 3.03)	0.369
		HIGH	1.61 (0.49, 5.31)	0.434
	Adjusted	LOW	Ref.	
		GREY	1.54 (0.69, 3.44)	0.293
		HIGH	2.13 (0.59, 7.68)	0.249
3-Year	Crude	LOW	Ref.	
		GREY	1.51 (0.70, 3.28)	0.294
		HIGH	2.23 (0.66, 7.53)	0.197
	Adjusted	LOW	Ref.	
		GREY	1.42 (0.68, 2.97)	0.354
		HIGH	1.57 (0.44, 5.53)	0.486

HR; Hazard Ratio, CI; Confidence Intervals

Supplement Table 5. Analysis of Banff i-IFTA (Inflammation in Areas of Interstitial Fibrosis and Tubular Atrophy) by AT1R-Ab Concentration Groups at 1-Year and 3-Year Follow-up

Follow-up period	Variable		HR(95% CI)	p-value
1-Year	Crude	LOW	Ref.	
		GREY	0.67 (0.20, 2.23)	0.513
		HIGH	0.65 (0.09, 4.81)	0.676
	Adjusted	LOW	Ref.	
		GREY	0.61 (0.18, 2.07)	0.424
		HIGH	0.49 (0.07, 3.63)	0.487
3-Year	Crude	LOW	Ref.	
		GREY	1.38 (0.38, 5.06)	0.624
		HIGH	1.50 (0.19, 11.65)	0.699
	Adjusted	LOW	Ref.	
		GREY	1.30 (0.32, 5.18)	0.714
		HIGH	1.18 (0.16, 9.02)	0.871

HR; Hazard Ratio, CI; Confidence Intervals

Supplement Table 6. Analysis of PTC (Peritubular Capillaritis) by AT1R-Ab Concentration Groups at 1-Year and 3-Year Follow-up

Follow-up period	Variable		HR(95% CI)	p-value
1-Year	Crude	LOW	Ref.	
		GREY	1.59 (0.57, 4.43)	0.376
		HIGH	4.77 (1.56, 14.55)	0.006
	Adjusted	LOW	Ref.	
		GREY	1.55 (0.56, 4.33)	0.402
		HIGH	4.43 (1.55, 12.67)	0.005
3-Year	Crude	LOW	Ref.	
		GREY	2.25 (0.75, 6.75)	0.148
		HIGH	7.80 (2.34, 25.95)	0.001
	Adjusted	LOW	Ref.	
		GREY	2.23 (0.75, 6.58)	0.147
		HIGH	7.49 (2.40, 23.40)	0.001

HR; Hazard Ratio, CI; Confidence Intervals

Supplement Table 7. Analysis of chronicity score by AT1R-Ab Concentration Groups at 1-Year and 3-Year Follow-up

Follow-up period	Variable		HR(95% CI)	p-value
1-Year	Crude	LOW	Ref.	
		GREY	0.75 (0.44, 1.28)	0.292
		HIGH	0.83 (0.34, 2.03)	0.676
	Adjusted	LOW	Ref.	
		GREY	0.74 (0.43, 1.28)	0.283
		HIGH	0.72 (0.27, 1.89)	0.501
3-Year	Crude	LOW	Ref.	
		GREY	1.04 (0.60, 1.78)	0.897
		HIGH	1.42 (0.57, 3.54)	0.455
	Adjusted	LOW	Ref.	
		GREY	1.03 (0.60, 1.76)	0.918
		HIGH	1.25 (0.48, 3.28)	0.655

HR; Hazard Ratio, CI; Confidence Intervals
